# Depth of submucosal invasion vs. Haggitt level as prognostic predictors of pedunculated‑type early‑stage colorectal cancer removed by endoscopic resection 

**DOI:** 10.3892/mi.2025.217

**Published:** 2025-02-05

**Authors:** Yasuo Imai, Yosuke Otake, Tomohide Tamachi, Tateki Yamane, Hitoshi Shimao, Shiro Sugihara, Masanori Ichinose

**Affiliations:** 1 Department of Diagnostic Pathology, Ota Memorial Hospital, SUBARU Health Insurance Society, Ota, Gunma 373-8585, Japan; 2 Department of Gastroenterology, Ota Memorial Hospital, SUBARU Health Insurance Society, Ota, Gunma 373-8585, Japan; 3 Department of Digestive Surgery, Shioya Hospital, International University of Health and Welfare, Yaita, Tochigi 329-2145, Japan; 4 Department of Gastroenterology, Shioya Hospital, International University of Health and Welfare, Yaita, Tochigi 329-2145, Japan

**Keywords:** early-stage colorectal cancer, endoscopic resection, pedunculated-type, depth of submucosal invasion, JSCCR, pT1b, Haggitt level, lymph node metastasis, recurrence, additional surgery

## Abstract

Additional surgery is considered when deep submucosal (SM) invasion (≥1,000 µm) is pathologically observed following the endoscopic resection of early-stage colorectal cancer (eCRC). The Japanese Society for Cancer of the Colon and Rectum (JSCCR) states that the depth of SM invasion be measured from the lower border of the muscularis mucosae (MM) when MM can be identified/estimated and from the lesion's surface when it cannot, irrespective of macroscopic types. In MM-tangled pedunculated eCRC cases, SM invasion depth should be measured from the reference line, which is the boundary between the tumor head and stalk. In the present study, in order to validate these measuring rules compared with the Haggitt classification, 73 consecutive pedunculated eCRC cases were analyzed. Using Elastica-van Gieson and desmin immunostaining, 37, 10, 18 and eight cases were diagnosed as pTis (without SM invasion by JSCCR)/Haggitt level (HL) 0, pTis/HL1-2 (head invasion), pT1b (SM invasion ≥1,000 µm)/HL1-2 and pT1b/HL3 (stalk invasion), respectively. No lymph node metastasis was observed. Recurrence occurred in one pTis/HL1 case; however, no pT1b/HL1-3 cases experienced recurrence. These results suggest that the pedunculated eCRC may be overstaged by the JSCCR rule in terms of adverse outcomes. The Haggitt classification, which evaluates HL1-3 as a shallow SM invasion, may be more useful when considering additional surgery.

## Introduction

Endoscopic resection is currently accepted as an appropriate treatment method for early-stage colorectal cancer (eCRC). Intramucosal CRC does not metastasize to the lymph nodes and can be curatively removed by endoscopic resection (1,2). By contrast, 6-12% of invasive eCRC cases, in which CRC invades through the muscularis mucosae (MM), but does not extend into the muscularis propria (MP), are associated with lymph node metastasis, thus requiring additional colectomy/proctectomy and lymph node dissection for curative treatment (3-6).

eCRC is grossly classified as a superficial type (type 0), which is further divided into protruded type (type 0-I) and superficial type (type 0-II) according to the Japanese Classification of Colorectal, Appendiceal, and Anal Carcinoma, third English edition, published in 2019 by the Japanese Society for Cancer of the Colon and Rectum (JSCCR). This is referred to as the JSCCR rule hereafter (7). The protruded type can be further subclassified as the pedunculated type (type 0-Ip), subpedunculated type (type 0-Isp), or sessile type (type 0-Is) from the presence or absence of a pedicle (7), while the Paris classification states that the subpedunculated type should be managed in the same manner as the sessile type (8). The JSCCR project study revealed that the nodal metastasis rate of eCRC with a submucosal (SM) invasion ≥1,000 µm was 12.5% (9). Additional surgery may be warranted in patients with a SM invasion ≥1,000 µm when other risk factors for lymph node metastasis are present, such as lymphovascular invasion, poorly or undifferentiated component (poorly differentiated adenocarcinoma, mucinous adenocarcinoma, signet-ring cell carcinoma), muconodules at the invasion front, or high grade of tumor budding/sprouting (BD) (10-12). The depth of SM invasion is simply determined in sessile type cases; however, it is relatively complex in the pedunculated type due to the presence of stalks and tangled MM. In Western countries, the Haggitt classification is used to determine the depth of SM invasion. This system classifies head invasion [Haggitt level (HL) 1 and HL2] and stalk invasion (HL3) as a shallow SM invasion that is equivalent to a SM invasion <1,000 µm in sessile lesions (13-15), while SM invasion of the underlying bowel wall (HL4) is recognized as a risk factor of synchronous lymph node metastasis and the need for additional surgical resection (16-19). By contrast, the JSCCR rule has stated that the depth of SM invasion should be measured from the MM lower border when the MM can be identified or estimated and from the surface of the lesion when it cannot be identified or estimated, irrespective of macroscopic types. In an MM-tangled pedunculated lesion, the depth of SM invasion should be measured from the reference line (7). With the JSCCR rule, additional surgery may be considered in some pedunculated eCRC cases with head and stalk invasion in which additional surgery can be spared by the Haggitt classification method.

The present study aimed to validate the JSCCR rule of measuring the SM depth in pedunculated-type eCRC cases, and to subsequently compare its utility in considering the need for additional surgery with that of the Haggitt classification method.

## Materials and methods

### Patients and study subjects

Consecutive patients with pedunculated-type eCRC who underwent endoscopic cold snare polypectomy or endoscopic mucosal resection at Shioya Hospital, International University of Health and Welfare (IUHW; Yaita, Japan) between 2006 and 2022 and Ota Memorial Hospital (OMH), SUBARU Health Insurance Society (Ota, Japan) between 2014 and 2022 were included in this study. The exclusion criteria included having a history of invasive CRC within 5 years of endoscopic resection, concurrent invasive CRCs that were resected soon after the endoscopic resection of pedunculated lesions, unavailable clinical information and surveillance <100 days following endoscopic resection. Any eCRC cases with HL ≥1 in which contrast-enhanced computed tomography (CECT) was not performed following endoscopic resection were also excluded. Clinicopathological information was obtained through the electronic chart systems of the respective institutions. When the adenocarcinoma was intraepithelial carcinoma or was confined within the lamina propria mucosae (LPM) and had negative resection margins, the patients were recommended to undergo a colonoscopy in 12 months. When the adenocarcinoma invaded beyond the MM, but had negative resection margins, the patients were followed-up every 6 months at the outpatient clinic for 5 years following endoscopic resection. Serum tumor marker levels, such as carcinoembryonic antigen and carbohydrate antigen 19-9, were measured every 6 months. CECT was performed immediately or after 6 months at the discretion of the clinicians and at 12 months following endoscopic resection. Colonoscopy was performed at 6 or 12 months following endoscopic resection at the discretion of the clinicians. In cases of carcinoma-positive resection margins, positive lymphovascular invasion and a SM invasion of ≥1,000 µm, additional surgery was considered. The present study was conducted in accordance with the Declaration of Helsinki, and the study protocol was approved by the Ethical Review Boards of the participating hospitals: IUHW, approval number: FK-94; OMH, approval number: OR24029. Due to the retrospective design of the study and anonymization of the data, consent to participate was not necessary.

### Pathological examination

**Figure 1:**
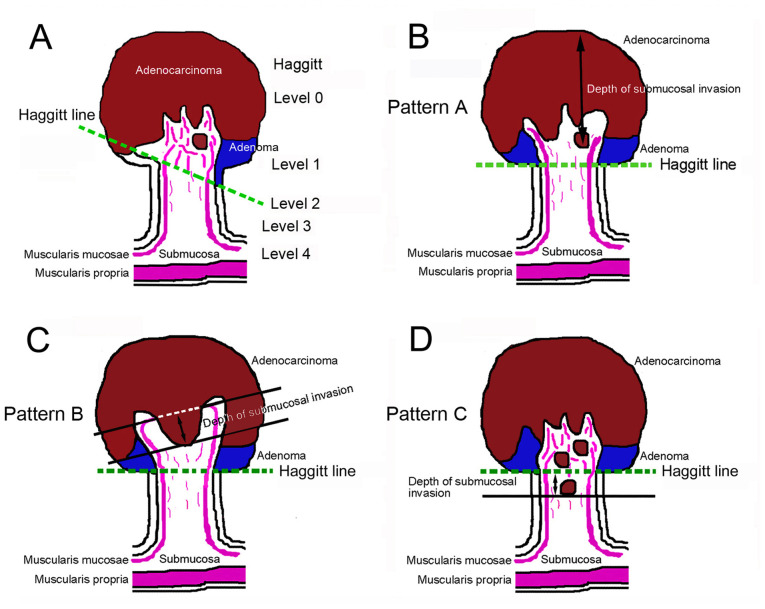
Method used for measuring the depth of SM invasion in pedunculated-type eCRC. (A) The Haggitt line, which links the junctions between the tumor and its stalk, was used to measure the depth of SM invasion rather than the reference line, which is defined by the JSCCR rule as the boundary between the tumor head and stalk. (B) When the MM could not be identified or estimated at the adenocarcinoma invasive front (designated as pattern A), the depth of SM invasion was measured from the lesion's surface. (C) If the MM could be identified or estimated in the original form (designated as pattern B), then the depth of SM invasion was measured from the lower border of MM. (D) When tangled MM was observed (designated as pattern C), depth of SM invasion was measured as the distance of invasion in the stalk beyond the Haggitt line. eCRC, early colorectal cancer; JSCCR, Japanese Society for Cancer of the Colon and Rectum; MM, muscularis mucosae; SM, submucosal.

All specimens were routinely processed for pathological diagnosis. The tumor size (maximal diameter), histological type and BD were evaluated using hematoxylin and eosin (H&E)-stained sections. Desmin protein staining was examined using immunohistochemistry (IHC) assays, as needed, to measure the distance of SM invasion. Elastica-van Gieson (EVG) staining and CD34 IHC assays, if necessary, were used to evaluate venous invasion, while D2-40 IHC assays were used to determine lymphatic invasion. Briefly, resected specimens were rapidly fixed in 10% neutral-buffered formalin solution for 24-48 h and embedded in a paraffin block. The blocks were then cut into 3-µm-thick sections for H&E and EVG staining, while they were cut into 4-µm-thick sections for IHC. Primary antibodies for IHC were FLEX monoclonal mouse anti-human desmin antibody (clone D33, 1:1; cat. no. IR606; Dako; Agilent Technologies, Inc.), mouse monoclonal anti-podoplanin antibody (clone D2-40, 1:1; cat. no. 760-4395; Ventana Medical Systems, Inc.), and CONFIRM™ anti-CD34 mouse monoclonal antibody (clone QBEnd/10, 1:1; cat. no. 790-2927; Ventana Medical Systems, Inc.). Antigen retrieval was performed as needed, by heating in Cell Conditioning Solution 1 (CC1) (Roche Diagnostics) at 100˚C for 60 min. Staining was performed on the VENTANA Benchmark XT automated slide stainer (Leica Microsystems GmbH) in combination with the *ultra*View DAB universal kit (Roche Diagnostics) according to the manufacturer's instructions. Nuclei were then counterstained with hematoxylin (Sakura Finetek Japan Co., Ltd.). Clinicopathological classification and staging were performed using the World Health Organization classification of colorectal tumors (5th edition) and the Union for International Cancer Control tumor, node, metastasis staging system (8th edition) (20,21). In addition, the subclassification of pathological tumor (pT)1 (the tumor is confined within the SM) was performed using the JSCCR rule with the following modifications: The Haggitt line, which links the junctions between the tumor and non-tumor in the stalk, was used to measure the depth of SM invasion instead of the reference line, which was defined by the JSCCR rule as the boundary between the tumor head and stalk (7) (Fig. 1A). pT1a and pT1b denote a SM invasion of <1,000 and ≥1,000 µm, respectively (7). BD was evaluated according to the JSCCR rule.

**Figure 2:**
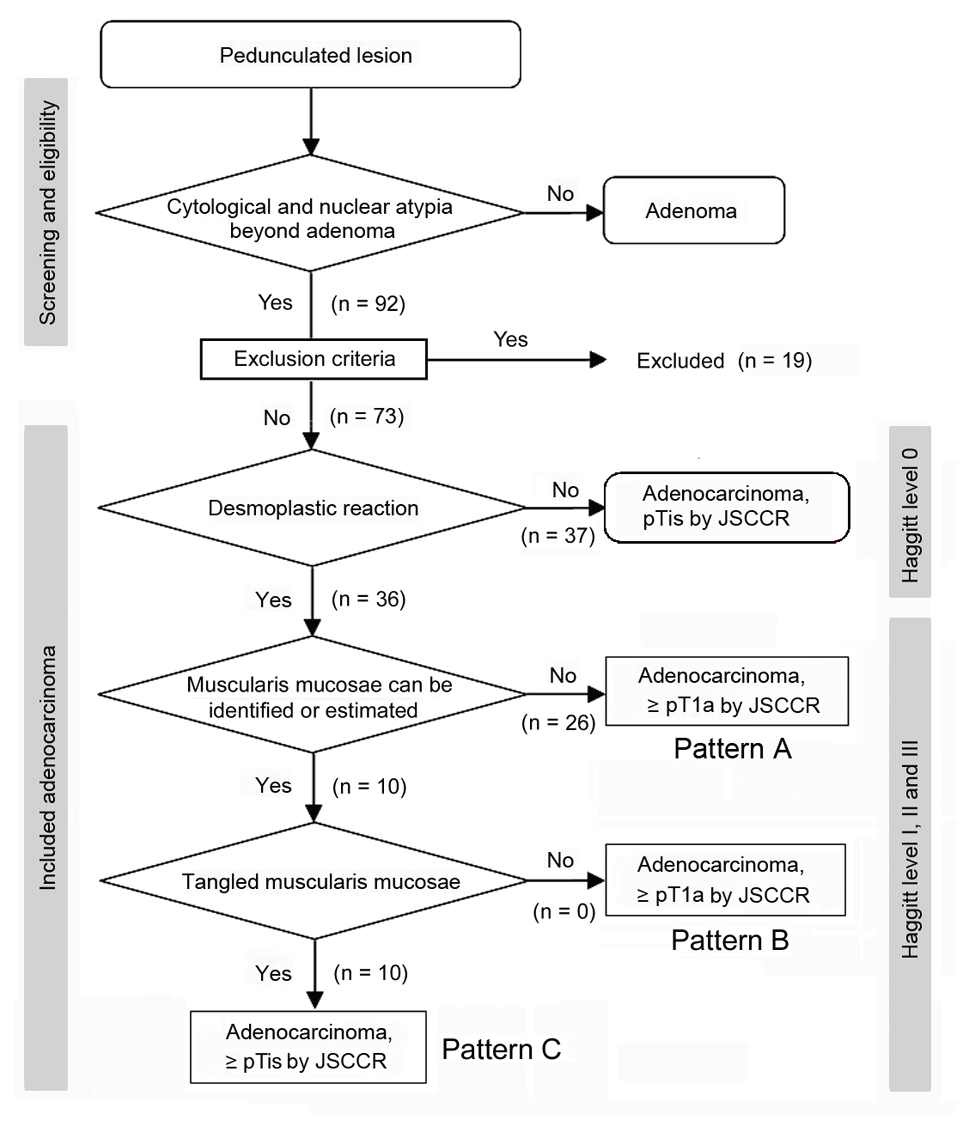
Flow diagram of the study subjects. JSCCR, Japanese Society for Cancer of the Colon and Rectum; pT1, pT confined within submucosa; pT1a, pT1 with a submucosal invasion of <1,000 µm; pTis, intraepithelial carcinoma or carcinoma confined within lamina propria mucosae.

**Figure 3:**
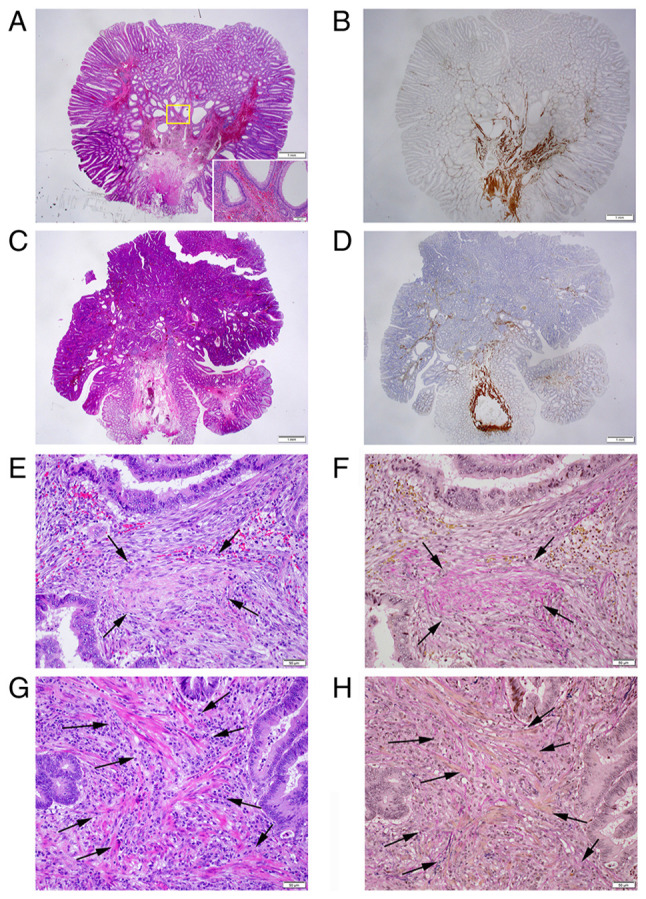
Pitfalls in cancer diagnosis, pT staging and discrimination between desmoplastic reaction and a tangled MM. (A) Representative histopathology of pedunculated-type tubular adenoma displaying SM pseudoinvasion of adenomatous glands (H&E; magnification, x1.25); Inset: Area within the yellow square. Pseudoinvasive glands lack cytological atypia strong enough for malignancy (H&E; magnification, x10). (B) Desmin IHC assays demonstrate a tangled MM in the pedunculated-type tubular adenoma (magnification, x1.25). (C) Pedunculated-type adenocarcinoma initially appearing to present with head invasion with disappearance of the MM. However, closer examination demonstrated no desmoplastic reaction (case no. 58; H&E; magnification, x1.25). (D) Desmin IHC assays demonstrated a tangled MM. This case was ultimately evaluated as pTis/HL0 (magnification, x1.25). (E) Desmoplastic reaction. Dull pink spindle cells, specifically immature fibroblasts, irregularly proliferate with an edematous and myxoid background and with collagen fibers deposited in the center (arrows) (H&E; magnification, x20). (F) Desmoplastic reaction foci with collagen fiber deposit are stained red using the EVG staining (arrows; magnification, x20). (G) Tangled MM. Clear pink spindle cells (arrows), specifically smooth muscle cells, run in thin bundles (H&E; magnification, x20). (H) Smooth muscle cell bundles (arrows) are stained yellow using the EVG staining (magnification, x20). EVG, Elastica-van Gieson; H&E, hematoxylin and eosin; HL, Haggitt level; IHC, immunohistochemistry; MM, muscularis mucosae; pT, pathological tumor; pTis, intraepithelial carcinoma or carcinoma confined within the lamina propria mucosae; SM, submucosal.

**Figure 4:**
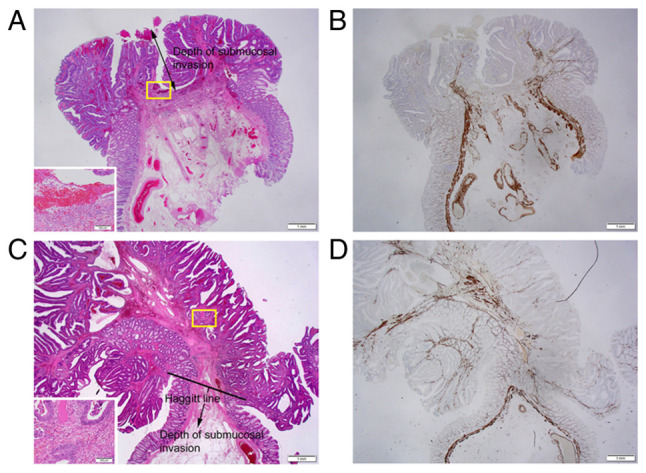
Representative histopathology of the MM pattern in pedunculated-type eCRC cases. (A) When the MM could not be identified or estimated at the adenocarcinoma invasive front (designated as pattern A), the depth of SM invasion was measured from the lesion's surface (H&E; magnification, x1.25); Inset: Area within the yellow square. Desmoplastic reaction at the tumor front, suggesting cancer invasion (H&E; magnification, x10; case no. 74). (B) Desmin IHC assays demonstrated the disappearance of the MM at the invasive front (magnification, x1.25). (C) When a tangled MM was observed at the adenocarcinoma invasive front (designated as pattern C), the depth of SM invasion was measured as the distance of invasion in the stalk beyond the Haggitt line (H&E; magnification, x1.25); Inset: Area within the yellow square. Desmoplastic reaction at the tumor front, suggesting cancer invasion (H&E; magnification, x10; case no. 41). (D) Desmin IHC assays demonstrated a tangled MM at the invasive front (magnification, x1.25). eCRC, early colorectal cancer; H&E, hematoxylin and eosin; IHC, immunohistochemistry; MM, muscularis mucosae; SM, submucosal.

The flow diagram of the pathological staging of pedunculated-type adenocarcinoma is presented in Fig. 2. Non-cancer glands often display SM misplacement or pseudoinvasion in pedunculated-type tumors. Therefore, submucosally placed glands were evaluated as pseudoinvasion when they did not display cytological or nuclear atypia strong enough for malignancy (Fig. 3A and B). In addition, the present study focused on desmoplastic reaction, with adenocarcinoma cases without desmoplastic reaction being diagnosed as intraepithelial carcinoma or carcinoma confined within the LPM, corresponding to HL0 and pTis by the JSCCR rule (22) (Fig. 3C and D). Adenocarcinoma cases with desmoplastic reaction were staged as HL ≥1, which may be equivalent to pTis or pT1 by the JSCCR rule (7). Identification of the MM is pivotal for measuring the depth of SM invasion and pT1 substaging by the JSCCR rule. Although the MM can be easily identified on the H&E- and EVG-stained sections by experienced pathologists (Fig. 3E to H), it may be sometimes difficult to distinguish between a tangled MM and carcinoma-associated fibrosis (23,24). Desmin IHC assays were performed in such cases. When the MM could not be identified or estimated at the adenocarcinoma invasive front (pattern A) (Fig. 1B), the depth of SM invasion was measured from the surface of the lesion (Fig. 4A and B). These cases were staged as ≥pT1a and HL ≥1. When the original MM could be identified or estimated, the depth of SM invasion was measured from the lower border of the MM (pattern B) (Fig. 1C). In such cases, the lesions were staged as ≥pT1a and HL ≥1. When a tangled MM was observed (pattern C) (Fig. 1D), the depth of SM invasion was measured as the distance of invasion in the stalk beyond the Haggitt line (Fig. 4C and D). The SM depth of head invasion is deemed to be <0 mm and the JSCCR rule specifies that such cases should be described only as head invasion. Thus, cases of head invasion with MM pattern C are practically treated as pTis. In the present study, cases with MM pattern C are described as ≥pTis and HL ≥1. Pathological diagnosis was independently performed by two experienced pathologists, with diagnostic discordance resolved by their discussion.

### Statistical analysis

Categorical variables between two groups were compared using the Fisher's exact test in the case of 2x2 cross tabulations or the Chi-squared test with or without Yates' correction as appropriate in the case of m x n cross tabulations. Continuous variables between two groups were compared using the Mann-Whitney U test. Continuous variables among three groups were compared using the Kruskal-Wallis H test, with the ad-hoc test performed by comparing two groups using the Mann-Whitney U test with the Bonferroni correction. P-values <0.05 were considered to indicate statistically significant differences, apart from the Mann-Whitney U test with the Bonferroni correction, for which the significance level was modified according to the number of groups. Survival curves were generated using the Kaplan-Meier method and significant differences in survival were analyzed using the log-rank test. Statistical analyses were performed using IBM SPSS Statistics 20 (IBM Corporation).

## Results

### Pathological diagnosis, evaluation of pT stage and HL

Overall, a total of 92 cases of pedunculated-type adenocarcinoma with/without adenoma component underwent endoscopic resection during the study period. Among these cases, 19 cases were excluded according to the exclusion criteria. A total of 73 cases, of which 70 cases had success with *en bloc* endoscopic resection, were subjected to further analysis. Of note, 1 of the 3 cases which failed with *en bloc* endoscopic resection underwent additional surgical resection. From the absence of desmoplastic reaction, 37 cases were diagnosed as intraepithelial carcinoma or carcinoma confined within the LPM, corresponding to pTis by the JSCCR rule and HL0. Cases of adenocarcinoma with desmoplastic reaction were classified as HL ≥1, which corresponds to pTis or pT1 by the JSCCR rule. Further pathological substaging was performed by measuring the depth of SM invasion. When the MM could not be identified or estimated at the adenocarcinoma invasive front (pattern A; Fig. 1B), the depth of SM invasion was measured from the surface of the lesion. This pattern was observed in 26 cases, which were staged as ≥pT1a and HL ≥1. When the MM could be identified or estimated in an original form at the adenocarcinoma invasive front, the depth of SM invasion was measured from the lower border of the MM (pattern B; Fig. 1C). This pattern, which represents ≥pT1a and HL ≥1, was not observed in any of the included cases. When a tangled MM was observed, the depth of SM invasion was measured as the distance of invasion in the stalk beyond the Haggitt line (pattern C; Fig. 1D). This pattern was observed in 10 cases, which were classified as HL ≥1, corresponding to the pTis (within the Haggitt line) or ≥pT1a (beyond the Haggitt line) stage by the JSCCR rule.

Ultimately, 47 cases were classified as pTis and 26 cases were classified as pT1b by the JSCCR rule. The pTis cases were significantly older than the pT1b cases (P=0.040). Of the pTis cases, 37 cases were HL0 (surveillance, 149-6,126 days; median, 2,106 days) and 10 cases were HL1-2 (surveillance, 112-2,860 days; median, 877 days). Of the pT1b cases, 18 cases were HL1-2 (surveillance, 104-4,079 days; median, 1,050 days) and 8 cases were HL3 (surveillance, 501-4,140 days; median, 1,306 days) (Table I).

The endoscopic resection margin status was unknown in 1 case of pTis/HL1, which was excised in two sections, with the patient later undergoing additional surgery. Additional surgical resection was performed in 1, 3 and 6 cases of pTis/HL1, pT1b/HL1, and pT1b/HL3, respectively. The incidence of additional surgery was significantly more frequent in the pT1b cases than in the pTis cases (P<0.001), and in the HL3 cases than in the HL0-2 cases (4/65 vs. 6/8: P<0.001). The clinicopathological characteristics of the the patients are summarized in Table I.

### Pathological features, pT stage and HL of the lesions

All but one of the cancer cases were well- or moderately differentiated adenocarcinoma, with the other case (pT1b/HL3) being mucinous adenocarcinoma. There was a significant difference in tumor diameter between pTis (0.9-36 mm; median, 6.0 mm) and pT1b (2-40 mm; median, 10.5 mm) cases (P<0.001), and among HL0 (0.9-14 mm; median, 4 mm), HL1-2 (2-36 mm; median, 10 mm) and HL3 (6-40 mm; median, 10.5 mm) cases (P<0.001). The post hoc analysis revealed that tumor diameter was significantly larger in HL1-2 (P<0.001) and HL3 (P=0.001) cases compared with HL0 cases. The absence of adenoma component was significantly more frequent in pT1b cases than in pTis cases (P=0.002) and in HL3 cases than in HL0 cases (P<0.001), although no significant difference was observed between HL1-2 and HL0 cases (P=0.570). The depth of SM invasion in pT1b cases, as defined by the JSCCR rule, was 2.0-15 mm (median, 5.0 mm) in 18 HL1-2 cases and 2.0-18 mm (median, 4.5 mm) in 8 HL3 cases. These results are summarized in Table II.

The lymphatic invasion rate was marginally more frequent in pT1b cases than in pTis cases (P=0.051), while the venous invasion rate was significantly more frequent in pT1b cases than in pTis cases (P=0.042). There was no significant difference in the lymphatic invasion rate and venous invasion rate among HL0, HL1-2 and HL3 cases, and BD between HL1-2 and HL3 cases (P=0.156, 0.632 and 0.999, respectively) (Table III).

### pT staging, HL and adverse outcomes

**Figure 5:**
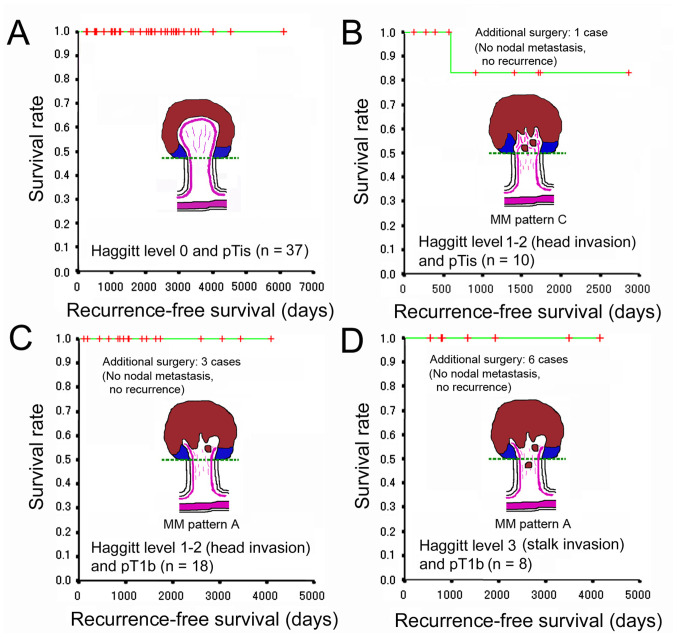
Recurrence-free survival of pedunculated-type eCRC cases following polypectomy. (A) pTis/HL0. (B) pTis/HL1-2 (head invasion) with MM pattern C. (C) pT1b/HL1-2 (head invasion) with MM pattern A. (D) pT1b/HL3 (stalk invasion) with MM pattern A. eCRC, early colorectal cancer; HL, Haggitt level; MM, muscularis mucosae; pT1b, deep submucosal invasion (≥1,000 µm); pTis, without submucosal invasion.

In the present study, an adverse outcome was defined as lymph node metastasis detected by the imaging studies and/or the pathological inspection of surgically resected specimens and recurrence diagnosed clinically and/or pathologically. Additional colectomy/proctectomy with lymph node dissection following endoscopic resection was considered in the cases with positive/unknown resection margins, lymphovascular invasion, BD2 (5-9 buddings/x200 field), and pT1b by the JSCCR rule. Due to such risk factors and the intentions of patients, additional surgery was performed in 1 case of pTis and 9 cases of pT1b. The inspection of the surgical specimens from those cases revealed no nodal metastasis. Recurrence was not observed in any of the pTis/HL0 cases. Of note, 1 case of pTis/HL1 experienced recurrence in the peritoneum 594 days following endoscopic *en bloc* resection with negative resection margins. The stalk of the lesion was 8 mm in diameter. The total size was 36x33 mm and the size of the adenocarcinoma was 10x10 mm. The histological type was moderately differentiated adenocarcinoma with MM pattern C in traditional serrated adenoma. No lymphatic or venous invasion was seen by D2-40 and EVG staining, respectively. Muconodules were observed at the invasive front, but the case was classified as pTis (head invasion) by the JSCCR rule. Recurrence was not observed in any of the pT1b/HL1-2 or pT1b/HL3 cases. The adverse outcome rates were 0, 3.6 and 0% in the HL0, HL1-2 and HL3 cases, respectively. No significant difference was observed in the recurrence rate and the recurrence-free survival between pTis and pT1b cases and among HL0, HL1-2, and HL3 cases. No CRC-related deaths were reported at the time of writing the present study. These results are presented in Fig. 5 and summarized in Table III.

## Discussion

In 2004, Kitajima *et al* (25) reported the results of the project study led by the JSCCR that analyzed 865 pT1 CRC cases that had been surgically resected. Lymph node metastasis was found in 87 cases (10.1%), comprising 10 (7.1%) of the 141 pedunculated lesions and 77 (10.6%) of the 724 non-pedunculated lesions. Lymph node metastasis was not observed in the pedunculated lesions with head invasion and stalk invasion presenting an SM depth <3,000 µm if lymphatic invasion was negative. The vertical distance from the Haggitt line to the deepest portion of invasion was designated as the SM depth in the pedunculated lesion. The nodal metastasis rate in the non-pedunculated lesions was 0% (0/123) when SM invasion was <1,000 µm and 12.8% (77/601) when SM invasion was ≥1,000 µm (25). From these results, the JSCCR guidelines 2005 for the treatment of CRC (26) stated that additional surgery following endoscopic resection of CRC should be considered when any of the following conditions was noted: i) Positive SM vertical margin; ii) SM invasion of ≥1,000 µm; iii) positive lymphatic and/or venous invasion; and iv) poorly differentiated adenocarcinoma and undifferentiated carcinoma. In these guidelines, unlike in the study by Kitajima *et al* (25), the depth of SM invasion was determined to be measured from the lower MM border when the MM could be identified or estimated and from the lesion's surface when the MM could not be identified or estimated, irrespective of the macroscopic type. In addition, the SM depth was to be measured from the reference line to the deepest portion of invasion in MM-tangled pedunculated lesions (26). Consequently, some cases with head invasion may be evaluated as pT1b with deep SM invasion, making them candidates for additional surgery.

In the present study, strict pathological diagnostic criteria were applied for pT staging. In pedunculated lesions, differentiation was required for the findings of SM localization of normal and adenoma glands, specifically, SM misplacement and SM pseudoinvasion. These phenomena may cause pT overstaging and result in underestimating the prognosis of pT1 carcinoma cases. The present study thus rigorously evaluated nuclear atypia as malignancy and diagnosed SM invasion only when desmoplastic reaction was observed at the invasive front, as cancer invasion in the LPM usually does not cause desmoplastic reaction (22). Distinction between desmoplastic reaction and a dissected or fragmented MM is usually not difficult for experienced pathologists. However, EVG staining and desmin IHC assays were conducted, as appropriate, in difficult cases. Reportedly, reproducibility is not high among pathologists when distinguishing between MM-tangled and non-tangled cases (coincidence rate: κ value of 0.55) (27). Desmin IHC staining was used to help distinguish between the two.

Following these pathological evaluations, the cases included in the present study were classified as follows: 37 pTis/HL0 cases, 10 pTis/HL1-2 cases, 18 pT1b/HL1−2 cases, and eight pT1b/HL3 cases. As the adenocarcinoma size increased, the pT stage and HL also increased, while the rate of coexisting adenoma decreased. As was expected, no adverse outcomes were found in the pTis/HL0 cases. Notably, no adverse outcome was observed in any of the 26 pT1b/HL1-3 cases, although the JSCCR guidelines assumed lymph node metastasis in ~10% of the endoscopically resected pT1b eCRC cases (26). Herein, the depth of SM invasion in the pT1b/HL1−2 and pT1b/HL3 cases ranged from 2.0-15 mm (median, 5.0 mm) and 2.0-18 mm (median, 4.5 mm), respectively, far exceeding the thickness of the normal colorectal wall (~3.3 mm). These data suggested that pT1b substaging using the JSCCR rule may not be useful as a tool for determining the need for additional surgery.

The latest JSCCR guidelines 2019 for the treatment of CRC weakly recommends additional bowel resection with lymph node dissection for pT1b pedunculated lesions, although other factors, such as histological subtype, lymphovascular invasion, high BD rate (BD2/3), and the wishes of patients, need to also be taken into consideration (9). Since the JSCCR guidelines 2005, a limited number of studies have validated the prognostic utility of the pT1b substaging system in the pedunculated lesions. In 2004, Ueno *et al* (11) measured the SM depth in the same manner as the JSCCR rule and investigated the association of SM depth with lymph node metastasis. However, they did not perform pT staging, combining the data of pedunculated and non-pedunculated lesions and analyzing them as one dataset. In 2016, Asayama *et al* (28) solely reported the prognostic significance of pT1b substaging using the present JSCCR rule. Adverse outcomes were also very rare in their cases. The lymph node metastasis rate was 3.9%, while the recurrence rate was 1.3% (Table IV). Their data also suggested that pT1b substaging using the JSCCR rule may not be useful as a tool for determining the need for additional surgery. Furthermore, accurate pathological pT staging is somewhat difficult. SM misplacement and pseudoinvasion of neoplastic glands may result in overstaging. Additionally, pathologists often disagree on whether the MM is tangled or not. Therefore, we propose to reconsider the definition of deep SM invasion (pT1b) in pedunculated lesions in the JSCCR rule.

In 1985, Haggitt *et al* (13) reported that no nodal metastasis was observed in 36 cases of pedunculated CRC with HL1-3, while one HL3 patient succumbed due to the disease. Several researchers have since reported the rates of adverse outcomes, such as lymph node metastasis, recurrence and disease-related mortality, in pedunculated eCRC cases that underwent surgical or endoscopical resection (11,13,25,28-30) (Table V). According to these previous studies and the present study, the lymph node metastasis rates were 2.3 and 7.3% in HL1-2 and HL3 cases, respectively, while the recurrence rates were 0.3 and 0.9% in HL1-2 and HL3, respectively. Although Kitajima *et al* (25) emphasized the importance of vascular invasion as a criterion of the need for additional surgery, Matsuda *et al* (29) reported that the incidence of nodal metastasis was 0 (0%) of 101 cases with head invasion, in which lymphatic and/or venous invasion was observed in 29 (28.7%) cases. They thereby concluded that pedunculated invasive eCRC can be managed by endoscopic treatment alone. These studies suggest that additional surgery may not be necessary for pedunculated CRC cases with HL1-2, irrespective of their vascular invasion status. Although there are a limited number of similar studies from Europe and the USA, the latest European (31) and American (32) guidelines suggest that only HL4 is a risk factor of lymph node metastasis.

In the present study, notably, recurrence was observed in 1 case of pTis/HL1 without lymphovascular invasion by pathological inspection. Both pT staging by the JSCCR rule and the Haggitt classification were unable to help predict recurrence in this case, emphasizing that there is no method which can be used to completely predict adverse outcomes. The estimated risk level by pathological inspection can only be used as a basis for deciding whether to perform additional resections. Despite its shortcomings, it can be considered that the Haggitt classification may be more useful than the JSCCR rule as it can help avoid any unnecessary additional surgical procedures.

Finally, there are some interesting studies on the SM depth as a prognostic factor in eCRC. According to the study ‘The stratification of risk factors for the metastasis of pT1b SM cancer (SM invasion more than 1,000 µm)’ by JSCCR (33), the incidence of lymph node metastasis was 1.4% in cases wherein only SM invasion depth did not satisfy the criteria for radical cure and where no other risk factors for metastasis were observed. On the other hand, even if surgery for T1 eCRC was carried out at first, the incidence of metastatic recurrence was 1.5% for colon and 4.2% for rectum (34). These studies suggest that surgery performed due to the presence of only deep SM invasion may have only restricted merit on prognosis.

The present study has some limitations. Firstly, the present study was retrospective and was performed at two institutions in the northern Kanto Region in Japan. Therefore, the numbers of events were small, with potential ethnic bias. Secondly, cases that were endoscopically resected were included, but those which underwent surgery without endoscopic resection were not. Therefore, the lymph node metastasis rate may have been underestimated.

In conclusion, using the JSCCR rule to evaluate pedunculated eCRC may cause pT1 overstaging and unnecessary additional surgery. Other reference lines for measuring the SM depth, such as the Haggitt line and MM of the underlying bowel wall, should be reconsidered. Otherwise, pT should be excluded from the risk assessment when considering additional surgery in pedunculated eCRC cases.

## Figures and Tables

**Table I:** Clinicopathologic characteristics of the included cases.

pT (JSCCR)/HL	pTis/HL0	pTis/HL1-2	pT1a/HL1-2	pT1a/HL3	pT1b/HL1-2	pT1b/HL1-2	pT1b/HL3	pT1b/HL3	P-value (P1/P2)
MM pattern^a^	NA	C	A and B	A, B, and C	A	B	A	B and C	NA
No. of cases	37	10	0	0	18	0	8	0	NA
Sex (M:F)	24:13	7:3	-	-	16:2	-	5:3	-	0.280/0.446
Age (median)	35-88(71)	46-77 (65.5)	-	-	39-78(62)	-	54-82(65)	-	0.040/0.087
Tumor site (C:A:T:D:S:R:unknown)	1:3:1:5:19:7:1	0:0:0:1:9:0:0	-	-	1:0:1:1:13:2:0	-	0:2:0:1:3:2:0	-	0.999/0.844
Endoscopic *en bloc* resection (yes:no)	36:1	8:2	-	-	18:0	-	8:0	-	0.548/0.946
ER status (ER0:ER1:ERX)	37:0:0	9:0:1	-	-	18:0:0	-	8:0:0	-	NA
Additional surgery (yes:no)	0:37	1:9	-	-	3:15	-	6:2	-	<0.001/<0.001
Surveillance after endoscopic resection (median) (days)	149-6,126 (2,106)	112-2,860(877)	-	-	104-4,079 (1,050)	-	501-4,140 (1,306)	-	0.363/0.063

^a^Theoretically, pTis/HL1-2 of MM patterns A and B, pT1a/HL1-2 and pT1b/HL1-2 of MM pattern C and pTis/HL3 of MM patterns A, B and C do not exist. A, ascending colon; C, cecum; D, descending colon; ER, endoscopic resection margin; ER0, negative for residual tumor following endoscopic treatment; ER1, positive for residual tumor following endoscopic treatment; ERX, indefinite for residual tumor following endoscopic treatment; F, female; HL, Haggitt level; JSCCR, Japanese Society for Cancer of the Colon and Rectum; M, male; MM, muscularis mucosae; NA, not applicable; P1, P-value between pTis and pT1b; P2, P-value among HL0, HL1-2, and HL3; pT, pathological tumor; pT1a, shallow submucosal invasion (<1,000 µm); pT1b, deep submucosal invasion (≥1,000 µm); pTis, without submucosal invasion; R, rectum; S, sigmoid colon; T, transverse colon.

**Table II:** Pathological features of the lesions.

pT (JSCCR)/HL	pTis/HL0	pTis/HL1-2	pT1b/HL1-2	pT1b/HL3	P-value (P1/P2)
MM pattern	NA	C	A	A	NA
No. of cases	37	10	18	8	NA
Size of cancer (median) (mm)	0.9-14 (4.0)	6-36 (9.5)	2-30 (10.5)	6-40 (10.5)	<0.001/<0.001
Histology (W/M:P:Muc)	37:0:0	10:0:0	18:0:0	7:0:1	NA
Coexisting adenoma (yes:no)	36:1	10:0	16:2	3:5	0.002/<0.001
Depth of SM invasion (median) (mm)	-	-	2.0-15 (5.0)	2.0-18 (4.5)	NA/0.955^a^
Invasion below the Haggitt line (median) (mm)	-	-	-	0.3-3.0 (1.3)	NA

^a^P2 between HL1-2 and HL3. HL, Haggitt level; JSCCR, Japanese Society for Cancer of the Colon and Rectum; MM, muscularis mucosae; Muc, mucinous adenocarcinoma; NA, not applicable; P, poorly differentiated adenocarcinoma; P1, P-value between pTis and pT1b; P2, P-value among HL0, HL1-2, and HL3; pT, pathological T; pT1b, deep submucosal invasion (≥1,000 µm); pTis, without submucosal invasion; SM, submucosal; W/M, well- and/or moderately differentiated adenocarcinoma.

**Table III:** pT staging, Haggitt levels and adverse outcomes of the lesions.

pT (JSCCR)/HL	pTis/HL0	pTis/HL1-2	pT1b/HL1-2	pT1b/HL3	P-value (P1/P2)
MM pattern	-	C	A	A	NA
No. of cases	37	10	18	8	NA
Lymphatic invasion (yes)	0	1	2	2	0.051/0.156
Venous invasion (yes)	0	0	2	1	0.042/0.632
Budding (BD1:BD2)	-	9:1	15:3	7:1	0.999/0.999^a^
Positive nodes (by pathology)	0/37	0/10 (0/1)	0/18 (0/3)	0/8 (0/6)	0.999 (0.999)/NA (NA)
Recurrence	0	1	0	0	0.999/0.982
Recurrence-free survival (median) (days)	149-6,126 (2,106)	112-2,860(748)	104-4,079 (1,050)	501-4,140 (1,306)	0.338/0.059

^a^P2 between HL1-2 and HL3. BD1, tumor budding of 0-4 per x200 field; BD2, tumor budding of 5-9 per x200 field; HL, Haggitt level; JSCCR, Japanese Society for Cancer of the Colon and Rectum; MM, muscularis mucosae; NA, not applicable; P1, P-value between pTis and pT1b; P2, P-value among HL0, HL1-2, and HL3; pT, pathological tumor; pT1b, deep submucosal invasion (≥1,000 µm); pTis, without submucosal invasion.

**Table IV:** Adverse outcomes of JSCCR pT1 pedunculated-type lesions in the study by Asayama *et al* (28) and the present study.

	Rate of LN metastasis (%)		Recurrence rate (%)		
Author(s), year of publication	pT1a	pT1b	pT1a	pT1b	(Refs.)
Asayama *et al*, 2016	1/100 (1.0)	3/76 (3.9)	0/100 (0.0)	1/76 (1.3)	(28)
Present study	-	0/26 (0.0)	-	0/26 (0.0)	
Total (%)	1/100 (1.0)	3/102 (2.9)	0/100 (0.0)	1/102 (1.0)	

JSCCR, Japanese Society for Cancer of the Colon and Rectum; LN, lymph node; pT1, submucosal invasion; pT1a, shallow submucosal invasion (<1,000 µm); pT1b, deep submucosal invasion (≥1,000 µm).

**Table V:** Adverse outcomes of pedunculated-type lesions according to the Haggitt level reported in the literature.

	Rate of LN metastasis (%)			Recurrence rate (%)			Dead of disease (%)			
Author(s), year of publication	HL0	HL1-2	HL3	HL0	HL1-2	HL3	HL0	HL1-2	HL3	(Refs.)
Haggitt *et al*, 1985	0/18 (0.0)	0/9 (0.0)	0/4 (0.0)	-	-	-	0/65 (0.0)	0/26 (0.0)	1/10 (10.0)	(13)
Ueno *et al*, 2004	-	0/42 (0.0)	6/24 (25.0)	-	-	-	-	-	-	(11)
Kitajima *et al*, 2004	-	3/53 (5.7)	7/88 (8.0)	-	-	-	-	-	-	(25)
Matsuda *et al*, 2011	-	0/101 (0.0)	8/129 (6.2)	-	0/219 (0.0)	1/121 (0.8)	-	-	-	(29)
Asayama *et al*, 2016	-	1/78 (1.3)	3/98 (3.1)	-	0/78 (0.0)	1/98 (1.0)	-	0/78 (0.0)	0/98 (0.0)	(28)
Kimura *et al*, 2016	-	4/30 (13.3)	5/46 (10.9)	-	-	-	-	-	-	(30)
Present study	0/37 (0.0)	0/28 (0.0)	0/8 (0.0)	0/37 (0.0)	1/28 (0.0)	0/8 (0.0)	0/37 (0.0)	0/28 (0.0)	0/8 (0.0)	
Total	0/55 (0.0)	8/341 (2.3)	29/397 (7.3)	0/37 (0.0)	1/325 (0.3)	2/227 (0.9)	0/102 (0.0)	0/132 (0.0)	1/116 (0.9)	

HL, Haggitt level; LN, lymph node.

## Data Availability

The data generated in the present study are included in the figures and/or tables of this article.
